# *Coniocybe* Ach. Revisited

**DOI:** 10.3390/jof10050363

**Published:** 2024-05-20

**Authors:** Stella G. Temu, Sanja Tibell, Donatha D. Tibuhwa, Leif Tibell

**Affiliations:** 1 Department of Organismal Biology, Uppsala University, 753 10 Uppsala, Sweden; temustellag@gmail.com (S.G.T.);; 2 Department of Molecular Biology and Biotechnology, University of Dar es Salaam, Dar es Salaam P.O. Box 35179, Tanzania; dtibuhwa@yahoo.co.uk

**Keywords:** taxonomy, molecular phylogeny, nomenclature, new species

## Abstract

Calicioids form a research field that has encompassed ascomycetous fungi with stalked ascomata similar to those of the lichen genus *Calicium*. Early generic circumscriptions of calicioid lichens and fungi were mainly based on morphological and secondary chemistry information. After the introduction of molecular data, taxonomy in the group has been reconsidered. Here, based on a broad geographical sampling, *Coniocybe* Ach. was revised using molecular and morphological features. Three loci (ITS, LSU and *rpb1*) were compared to infer its phylogenetic position, and a total of 52 new sequences (14 ITS, 24 LSU and 14 *rpb1*) were produced. Apart from its type *C. furfuracea*, *Coniocybe* was revised and emended to also include *C. brachypoda* and *C*. *confusa*. In addition, a new species, *Coniocybe eufuracea*, was described, and a key to the species of *Coniocybe* was provided.

## 1. Introduction

Calicioid fungi has long been a research field of considerable interest. It includes the systematics of fungi having ascomata similar to those of *Calicium* Pers., i.e., stalked ascomata with a distinct head.

The investigation of calicioids dates back to the seminal papers of Acharius 1815–1817 [1,2,3] and has been further pursued by distinguished lichenologists such as Vainio 1927 [4], Keissler 1936–1938 [5] and Nádvorník 1942a, 1942b [6,7]. Only with the works of Schmidt 1970 [8] did it become obvious that calicioids were quite a heterogenous assemblage, both in morphology and ecology, and that although the majority were lichenized and mazaediate (i.e., having prototunicate asci and passive spore dispersal), others were not. The diversity of calicioids was further exposed by Tibell 1984 [9]. Genetic data subsequently substantiated these observations and showed calicioids to have a variety of phylogenetic origins. In consequence, a number of only distantly related higher taxa were recognized [9,10,11,12], Coniocybales being one of them. Coniocybaceae Rchb. in Coniocybales [9] comprise about 30 species in two genera, *Chaenotheca* (Th. Fr.) Th. Fr. and *Sclerophora* Chevall. [11,13]. *Sclerophora* was earlier treated in a separate family Sclerophoraceae [9]. *Chaenotheca* are crustose lichens with stalked apothecia [14] and (mostly) non-septate, spherical to ellipsoidal pale brown to brown spores.

*Coniocybe* Ach.: Fr. was described by Acharius [2]. The genus in Acharius’ circumscription included *Mucor furfuraceum (=C. furfuracea* (L.) Ach.)—in fact the only crustose calicioid described by Linnaeus—and also *C. stilbea* Ach. (an illegitimate name [15]), *C. brachypoda* Ach. [2] and *C. gracilenta* (Ach.) Ach. *Coniocybe* was accepted by E. Fries [16] and also by Th. M. Fries [17] and Zahlbruckner [18], as in contrast to *Chaenotheca* having a poorly developed excipulum. For many decades the genus was maintained until a major revision of the taxonomy of calicioids were undertaken by Tibell [9]. Taxonomy at that time relied mostly on morphologic and chemistry data, and, based on this, *Coniocybe brachypoda* and *C. furfuracea* were transferred to *Chaenotheca*. This wider concept of *Chaenotheca* will henceforth be called *Chaenotheca* s. lat. Somewhat later, a species very similar to *C. furfuracea*, was described under the name *Chaenotheca confusa* Tibell [19].

Molecular studies have proven increasingly important in elucidating taxonomic relationships of calicioid lichens by inference of their phylogeny [11,13]. In a recent study based on Internal Transcribed Spacer (ITS) sequences, it was shown that, within *Chaenotheca* s. lat., there are well characterized clades [20] which were also given preliminary nicknames. Thus *C. brachypoda* and *C. furfuracea* were shown to belong to a group referred to as ‘*Coniocybe* s. str.’. The species at that time included have slender, yellow-pruinose ascomata and are associated with *Stichococcus* sp. That study was limited to comparisons of species mainly from Europe. The inclusion of *C. brachypoda*, *C. furfuracea* and *C. confusa* in a resurrected *Coniocybe* as proposed below is a first step towards re-evaluating the taxonomy of *Chaenotheca* s. lat., in a context of a wider sampling of material.

The main focus of this study is the emendation of *Coniocybe* utilizing a combination of molecular and morphological features based on a wide geographical sampling.

## 2. Materials and Methods

### 2.1. Taxon Sampling

This study is partly based on African materials collected jointly by the authors in the Kilimanjaro and Arusha regions in 2018, while some other material was collected in Australia (Tasmania), in addition to a wide geographical representation of the species, mainly vouchered by herbarium material kept in UPS.

### 2.2. Morphological Features

Ascomata anatomy was observed on freezing microtome sections 10 to 15 μm thick and on squash preparations under the light microscope. The sections were mounted in water. The ascospores of some specimens were investigated under the scanning electron microscope (SEM).

When statistical estimates of sizes are given the following format is applied: mean (X) minus one standard deviation/(sd)/mean plus one standard deviation, number of observations (n), number of specimens observed (c). Unless otherwise stated, the number of observations is 40.

### 2.3. Molecular Methods

Total DNA was extracted from freshly collected material, or material temporarily kept at −20 °C by using the DNeasy Plant Mini Kit (Quiagen, Hilden, Germany), following the manufacturer’s instructions. About 10 to 30 apothecia were carefully selected under a dissecting microscope, cleaned from foreign material and used for DNA extraction. Total DNA was used for PCR amplifications with the following primers ITS1F [21], ITS4 [22] for ITS; LROR and LR5 [23] for the partial 28S large subunit rDNA (LSU), and gRPB1-A and gRPB1-C for the partial RNA polymerase II largest subunit RPB1 (*rpb1*) [24]. The AccuPower PCR PreMix (Bioneer, Daejeon, Republic of Korea) was used, adding 3 µL diluted DNA, 1.5 mL of each primer (10 mM), and water to a total volume of 20 µL. The PCR conditions for ITS and LSU were: initial denaturation for 4 min at 95 °C, followed by 35 cycles of 1 min at 94 °C, 1 min at 54 °C, 45 s. at 72 °C, and final elongation for 5 min at 72 °C. For *rpb1*, PCR amplifications were carried out using Illustra Hot Start PCR beads under the same PCR conditions. PCR products were visualized by electrophoresis on 1.5% agarose gels. Products were purified using Illustra™ (GE Healthcare UK Limited, Little Chalfont, UK) ExoStar buffer diluted 10×, following the manufacturer’s protocol. Sequencing was conducted by Macrogen (www.macrogen.com [25]. After assessment of their quality, the sequences were aligned by using MAFFT v7 (on-line server: https://mafft.cbrc.jp/alignment/server/, accessed on 14 May 2024), with G-INS-1 Strategy (Slow; progressive method) and default parameters.

The study involved two datasets. The larger dataset consists of three marker region (ITS, LSU, *rpb1*) sequences representing 15 species of *Chaenotheca* s. lat. (with newly produced sequences in bold, Table 1). They represented the clades already demonstrated and nicknamed by Tibell et al. [20]. *Sclerophora farinacea* was chosen as outgroup for the analyses. The second dataset, a smaller dataset, only included sequences for the species of *Coniocybe* s. str., and here *Chaenotheca biesboschii* was chosen as outgroup.

For both datasets, phylogenetic relationships and their posterior probabilities (PP) were inferred using a Bayesian approach, and additional support values were estimated using Maximum Likelihood Bootstrap Support (MLbs). For the Bayesian analyses, the most likely models of evolution were estimated using the Akaike Information Criterion (AIC) as implemented in Modeltest 3.7 [26]. For the first dataset, the GTR + I + G model of evolution was employed for ITS and LSU, and HKY + I + G was used for *rpb1*. For the second dataset, the GTR + G model was implemented for ITS. A conflict among single-locus datasets was considered significant if a well-supported monophyletic group (posterior probability [PP] ≥0.95) was found to be well supported as non-monophyletic when different loci were used. Further analyses were performed after concatenation using SequenceMatrix v1.8.2 [27].

The Bayesian analysis was executed using MrBayes v3.2.6 [28], where two analyses of two parallel runs were carried out for 10 M generations. Each run included four chains, and trees were sampled every 1000 generations and 25% were discarded as burn-in. All runs converged on the same average likelihood score and topology. Maximum Likelihood (ML) estimates were carried out by RAxML v8.2.10 using the GTR + G + I model of site substitution [29]. The branch support was acquired by maximum likelihood bootstrapping (MLbs) of 1000 replicates [30], and MLbs ≥ 70% were considered to be significant. The trees were visualized in FigTree v1.3.1 [31].

## 3. Results

### 3.1. Phylogeny of Chaenotheca *s. lat.*

**Figure 1:**
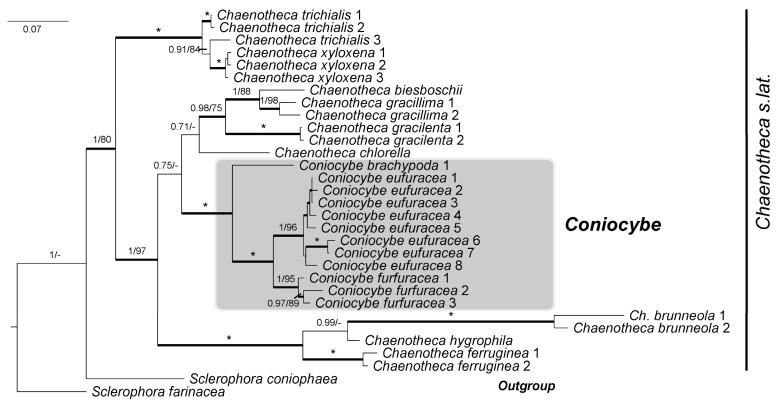
Consensus tree based on a Bayesian and Maximum Likelihood (ML) analysis of concatenated ITS, nuLSU and *rpb1* of *Chaenotheca* s. lat. showing the phylogenetic position of *Coniocybe*. The tree was rooted using *Sclerophora farinacea* and *S. coniophaea.* The two support values associated with each internal branch correspond to posterior probability (PP) and bootstrap support (bs), respectively. Branches in bold indicate a support of PP ≥ 95% and an MLbs ≥ 70%. An asterisk on a bold branch indicates that this node has a support of 100% for both support estimates. A dash instead of an MLbs value indicates that the node of the Bayesian tree was not recovered by ML bootstrapping. *Coniocybe* is highlighted by a shaded box.

A phylogeny of *Chaenotheca* s. lat., based on concatenation of the three loci of species representing the different clades provisionally named in Tibell et al. [20], is presented below (Figure 1). The analyses included 12 species of *Chaenotheca* s. lat. There was no conflict among the trees obtained for the individual locus (see Supplementary Materials; Figure S1: ITS phylogeny, Figure S2: LSU phylogeny, and Figure S3: RPB1 phylogeny).

In this phylogeny, *Coniocybe* is distinct from the clades of *Chaenotheca* s. lat. and it has maximum support in Bayesian and ML analyses. It is close to the ‘gracillima group’ in agreement with the results of Tibell et al. [20].

### 3.2. Phylogeny of Coniocybe

**Figure 2:**
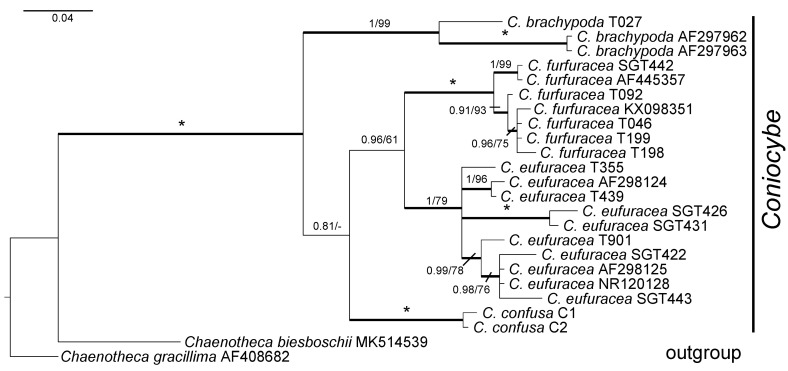
Phylogenetic relationships of 4 species of *Coniocybe* based on a Bayesian and Maximum Likelihood (ML) analysis of an ITS dataset. The tree was rooted using *Chaenotheca biesboschii* and *Chaenotheca gracillima*. The two support values associated with each internal branch correspond to posterior probabilities (PP) and maximum likelihood bootstrap support (MLbs) proportions, respectively. Branches in bold indicate a support of PP ≥ 95% and MLbs ≥ 70%. An asterisk on a bold branch indicates that this node has a support of 100% for both support estimates.

A phylogeny of *Coniocybe* is presented in Figure 2. The analysis includes sequences representing four species of *Coniocybe*, two of which (*C. brachypoda*, *C. furfuracea*) were shown to belong in ‘*Coniocybe* s. str.’ in Tibell et al. [20]. In addition two further species belong to *Coniocybe*, viz. *C. confusa*, that is sequenced here for the first time, and *C. eufuracea*, newly described here. *Coniocybe furfuracea* is a species with a wide distribution occurring on several continents. It has a characteristic spore ornamentation of reticulate ridges as investigated by scanning electron microscopy. The Tanzanian collections SGT 426 and SGT 431 of *C. eufuracea* are genetically very similar (Figure 2), but differ slightly from other collections of the species. However, a much wider sampling from all parts of the distribution area is required for resolving relationships within this species (or possibly species complex) and both molecular data, secondary chemistry and morphology need to be chartered in detail, which is beyond the scope of this investigation.

### 3.3. Taxonomy

***Coniocybe*** Ach. nom. sanct., emend. Temu & Tibell

*Coniocybe* Ach., K. Vetensk-Acad. Nya Handl. 4: 285 (1816).

Lectotype: *C. brachypoda* Ach. (Fink Cont. United States Nat. Herb. 14,1: 45 (1910)

**Figure 3:**
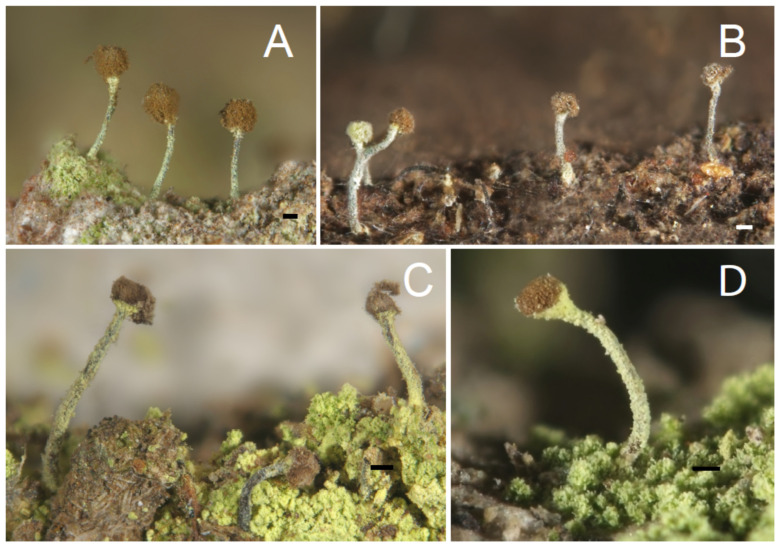
Ascomata of *Coniocybe* species; scales: 1 mm. (**A**): *C. eufuracea* (Temu 422); (**B**): *C. brachypoda* (Tibell 17062); (**C**): *C. confusa* (Kantvilas 280/19); (**D**): *C. furfuracea* (Temu 442). Pictures by George Hillman.

**Figure 4:**
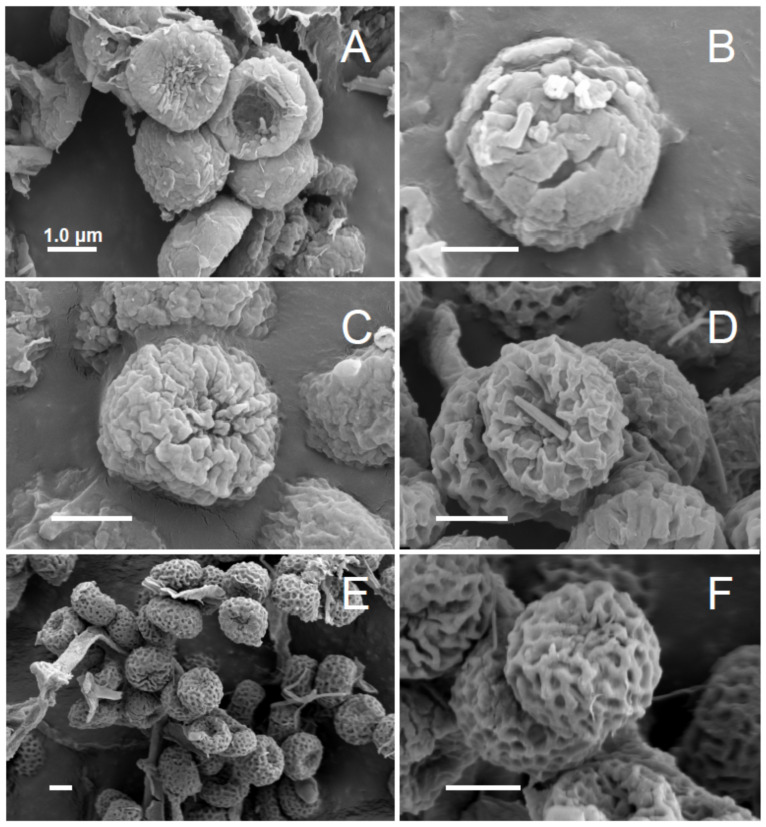
Spore ornamentations of *Coniocybe* species, SEM; scales: 1 µm. (**A**): minute irregularly arranged ridges of *C. brachypoda*; (**B**): irregular cracks of *C. brachypoda*; (**C**): short irregularly arranged ridges of *C. confusa*; (**D**): reticulate arranged ridges in *C. furfuracea*; (**E**,**F**): ornamentation of reticulate ridges of *C. eufuracea*.

Thallus crustaceous; ascomata with long stalks and rounded capitula (Figure 3) with inconspicuous or missing excipulum; asci catenulate, with croziers; spores spherical, non-septate, small, pale brown, with an ornamentation of minute irregularly arranged ridges (Figure 4); mazaedium well developed; secondary metabolites vulpinic acid derivatives; photobiont *Stichococcus* sp.

Apart from *C. brachypoda* Ach. and *C. furfuracea* (L.) Ach, *C. confusa* (Tibell) Temu & Tibell was found to belong here based on molecular information. One new species, *C. eufuracea* is here described.

Key to the species of *Coniocybe*
1.1 Apothecia 0.4–1.4 mm high, mazaedium medium brown*C. brachypoda*1.2. Apothecia 0.6–3.0 mm high, mazedium pale brown22.1. Spore surface with short, irregular ridges and cracks visible under the light microscope*C. confusa*2.2. Spores with reticulate ridges, but without cracks 33.1. Stalk 0.04–0.08 mm wide, spores 2.3–2.6 um diam.; 8–10 ornamentation ridges over the hemisphere; diagnostic sequence ITS1: 8–10 ornamentation ridges over the hemisphere; ITS1 diagnostic sequence: CTTCT; ITS2 diagnostic sequence: TGCAGC *C. eufuracea*3.2. Stalk 0.06–0.10 mm wide, spores 2.3–3.0 um diam; 5–6 ornamentation ridges over the hemisphere; ITS1 diagnostic sequence: TCGTGC; ITS2 diagnostic sequence: TGTAGT*C. furfuracea*

***Coniocybe brachypoda*** Ach.

*Coniocybe brachypoda* Ach., K. Vetensk Acad. Handl. 1816: 287 (1816).

Type (H-Ach 535, lectotype, Tibell, Symb. Bot. Ups. 27(1): 71, 1987).

Thallus immersed; apothecia short, 0.4–1.4 mm high, covered by a dense greenish pruina; mazaedium dark to medium brown, ± pruinose; capitulum spherical, 0.1–0.2 mm diam., with poorly developed excipulum; stalk 0.04–0.08 mm wide, covered with pruina; spores medium brown, spherical to somewhat cuboid, 3.0–4.5 µm diam., with a very minute ornamentation of tiny ridges and conspicuous, larger irregular cracks (Figure 4A,B); photobiont: *Stichococcus* sp.

Figure 3B and Figure 4A,B.

*Note*: Characterised by having rather short apothecia, an unusually immersed thallus and a rather dark brown mazaedium with, at least in young stages, a yellowish green pruina covering the mazaedium. Capitulum 0.1–0.2 mm diam. The spores are spherical to cuboid, 3–4.5 μm diam. and have an ornamentation of minute, irregularly arranged ridges not visible under the light microscope and larger, irregular cracks with SEM (Figure 4B) that are well within the resolution of the light microscope.

The images of *C. brachypoda* in Tibell [32] with Figure 44, agree well with this insofar that in the transmission electron microscopy image, while Figure 44A shows gaps in the outermost spore wall corresponding to cracks visible in our SEM view (Figure 4B), while the ridge ornamentation in Figure 44B is minute and only barely discernible. These then most likely represent *C. brachypoda*. However, for the New Zealand material, the thallus was described as episubstratic and green [32], which might indicate that at least some of the material used for the description in fact refers to misidentified *C. confusa*. *Coniocybe brachypoda* grows on bark and wood in shaded and humid situations. A very widely distributed species in the Northern Hemisphere and also known from New Zealand, whereas Australian [32] and South American [19] reports have not yet been supported by sequence data.

Selected specimens examined: Sweden, Jämtland, Kall par., 2 km NW of Kall, Sandnäset, between Stortjärnen and Svarttjärnen, Tibell, 1987, 17062 (UPS: GB: AF297962). Åre par. 10 km ESE of Handöl, 1 km from the mouth of River Järpån, Tibell 22193 (UPS; GB: AF297963). New Zealand, North Island, Tongariro National Park, 5.5 km NE of Ohakune Railway Station, 1986, Tibell 16627; UPSC2070 (UPS; GB: PP741625).

***Coniocybe confusa*** (Tibell) Tibell & Temu, comb. nov.

*Chaenotheca confusa* Tibell, Bibl. Lichenologica. 71: 46 (1998).

Holotype: Chile, Region XII, Isla Navarino, c 20 km E of Puerto Williams, c. 2 km SE of Puerto Eugenia, 1989, Tibell 17940 (UPS). MB: 853892.

Thallus superficial and well developed, farinose to minutely granular, yellowish green; apothecia long and slender, 2.3–3.0 mm high, covered by a dense greenish pruina; mazaedium pale brown, ± pruinose; capitulum spherical, 0.3–0.4 mm diam, with poorly developed excipulum forming a small collar at the base when young, covered by numerous hair-like crystals; stalk 0.10–0.15 mm wide, pruinose; Spores spherical, 2.5–3.5 µm diam. with an ornamentation of minute ridges and provided with distinct cracks visible under the light microscope ([19] with Figure 10E) and under SEM (Figure 4C); photobiont: *Stichococcus* sp.

Figure 3C and Figure 4C (see also Tibell [19] with Figure 10E).

Habitat. On tree trunks and decorticated stumps in dark and humid situations.

Distribution. Widely distributed in the Southern Hemisphere. Vouchered by molecular data from specimen from Australia.

*Note*. Characterized by having a farinose to minutely granular, greenish yellow thallus; long-stalked apothecia covered by a greenish-yellow pruina; a hemispherical to almost spherical capitulum with poorly developed excipulum; catenulate asci; and spherical to cuboid spores 2.5–3.5 µm diam. having a minutely fissured surface. Very similar to *C. furfuracea*, but differing in having higher ascomata, larger capitula and larger spores provided with distinct cracks visible under the light microscope. Known from temperate South America and Australasia.

Specimen examined: Australia, Tasmania, Eldon Road, alt. 300 m., 2019 Kantvilas 280/19, HO 598335; GB: PP741626, PP741627).

***Coniocybe eufuracea*** Temu & Tibell sp. nov.

Holotype: Tanzania, Arusha, Mt. Meru, 3°16′58.35″ S 36°42′09.41″ E, alt. 2096 m, on *Aguru salicifolia*, Temu 422 (UPS); GB: PP741593 (ITS); PP741622 (LSU); PP750715 (RPB1). MB: MB853735.

Thallus superficial and well developed, yellowish green; apothecia middle sized (Figure 3A), 0.6–1.5 mm high (X = 1.05 mm, sd = 0.45 mm, n = 40, c = 4), covered by a dense greenish pruina; mazaedium pale brown, ± pruinose; capitulum spherical, 0.16–0.22 mm diam., (X = 0.16 mm, sd = 0,03 mm, n = 40, c = 4) with poorly developed excipulum; stalk 0.04–0.08 mm wide (X = 0.06 mm, sd = 0.02 mm, n = 40, c = 4), pruinose; spores pale brown spherical, 2.3–2.6 μm diam. (X = 2.42 μm, sd = 0.13 μm, n = 40, c = 4) with a minute ornamentation of reticulate ridges (Figure 4E,F), 8–10 ornamentation ridges over the hemisphere; photobiont: *Stichococcus* sp.

Figure 3A and Figure 4E,F.

Habitat. On tree trunks and decorticated stumps in dark and humid situations.

Distribution. Widely distributed in cool temperate to temperate areas of the Northern Hemisphere. Vouchered by molecular data from specimens from Canada, India, Japan, Sweden and high altitude in Tanzania.

*Note*: Together with *Coniocybe confusa* and *C. furfuracea* forming a complex of (macro-) morphologically cryptic species that differ in the DNA of the ITS and LSU regions. *C. eufuracea* differs from *C. furfuracea* in having shorter apothecia, wider stalks and smaller spores with an ornamentation of 8–10 ridges over the hemisphere, with small interstices. It differs from *C. confusa* in having shorter apothecia, a smaller capitulum and smaller spores with an ornamentation of 8–10 ridges over the hemisphere, with smaller interstices but no coarse cracks (Figure 4E,F).

Additional specimens examined: Canada, Kouchibouguad National Park, S bank of Black river N of the Biodiversity monitoring site, 46:50N 65:00:33W, 005m, 2001, on decayed wood of *Betula alleghaniensis*, Koffman 387 (UPS; GB: PP741591, PP741620). **India**, Uttaranchal, 25.5 km NNE of Ghuttu, above Kharsoli, on the W valley slope, in mixed *Picea-Quercus semecarpifolia* forest, on decorticated stump of *Q. semecarpifolia*, 30°44′ N, 78°53′ E, 2003, Tibell 23224 (UPS; GB: PP741628), 20 km NNE of Uttarkashi, Dodital, 2008 Tibell 25024 (UPS); Ghangaria just S of the village on W-facing slope, 2008 Tibell 25106 (UPS; GB: PP741594, PP741621, PP750719). Japan, Honshu, Kanagawa Pref. (Sagami Prov.), Odawara city, 80 km SW of Tokyo, 4 km ESE of the town Odawara, 100–400 m N of the 300-year-old cherry tree Shidare-zakura, 1 km NW of Iryuda railway station, deciduous forest along small path up in the mountains, on deciduous tree, 35°15′ N, 139°07′ E, 200 m, Thor 15698 (hb. Thor; GB: AF298124). Sweden, Uppsala, Fiby Urskog, 59°53′ N 17°20′ E, 46 m, 2020, Temu 443 (UPS; GB: PP741592, PP741623, PP750717); Jämtland, Åre par., 10 km ESE of Handöl, 1 km from the mouth of River Järpån, 1999, Tibell 22190 (UPS; GB; AF298125). Tanzania, Kilimanjaro Region, Kilimanjaro, Moshi, Mweka Route, 03°10′ S, 37°21′ E, 2700–2900 m, at base of old *Podocarpus* in podocarp mountain forest, Temu 431 (UDSM; GB: PP741590, PP741617, PP750716); Kilimanjaro National Park, Marangu route, 3°05′ S 37°10′ E, 2718 m., Temu 426 (UDSM; GB: PP741589, PP741616). Location unknown: Wedin 6366 (UPS, GB: NR120128).

***Coniocybe furfuracea*** (L.) Ach.

Kongelige Svenska Vetensk. Akad. Handl.: 1816: 286.

*Mucor furfuraceus L.*, *Sp. pl.* **2**: 1185 (1753). Epitype proposed here: Uppland, Dannemora par., 0.5 km S of Ruddu, 2000, Tibell 22364 (UPS; GB AF445357; MB Typification Number: 10020138).

*Nomenclatural note*: There is no material of *Mucor furfuraceus* in the Linnaean herbarium [33]. In this paper, there also is a claim that a neotype was designated. However, no identification information was given for this alleged neotype. In the lichenological tradition, *C. furfuracea* has since long been recognized as a widely distributed and in many areas fairly common and easily recognized species. *Coniocybe furfuracea* was included in *Chaenotheca* as *Chaenotheca furfuracea* (L.) Tibell [9], although the inclusion of *Coniocybe* in *Chaenotheca* was described as provisional. As shown here, *Coniocybe*, in a three-marker phylogeny, is clearly within *Chaenotheca* s. lat. sensu Tibell [9], but also monophyletic and distinct, both in the DNA regions applied and in morphology. Here, we have shown that in an emended and resurrected *Coniocybe*, a complex of three morphologically cryptic species occur, two of them in Europe, viz. *C. furfuracea* and the newly described *C. eufuracea*, its sister species. To resolve the nomenclatural situation of *C. furfuracea* an epitypification is suggested. This is not without complication, since this species in Acharius’sense might just as well have been *C. eufuracea*, but, until our suggestion has been proven wrong, we find the suggested epitypification a reasonable tentative solution. In the protologue, Solander was given as the collector and we find it suitable to epitypify based on a recent Swedish collection for which some molecular information is available.

Thallus superficial, farinaceous, intensely yellowish green, occasionally almost completely immersed; apothecia tall, 1.6–2.6 mm high, mazaedium pale brown, ±pruinose; capitulum spherical, 0.1–0.2 mm in diam., with a poorly developed or lacking excipulum; stalk 0.06–0.10 mm diam., covered by a dense yellowish green pruina; spores pale brown. spherical, 2.3–3.0 µm diam., with an ornamentation of reticulate ridges just discernable under the light microscope. Photobiont *Stichococcus* sp.

Chemistry. Thallus K-, C-, KC-, PD-. The thallus contains vulpinic acid, pulvinic acid and pulvinic dilactone, substances which also form the pruina of the ascomata.

Habitat. In dark and humid situations, particularly on rootlets and soil of uprooted trees and decorticated stumps in coniferous forests, more rarely on deciduous trees.

Distribution. Wide distribution in cool temperate to temperate areas of the Northern Hemisphere (Eurasia, North America). Vouchered by molecular data from specimens from India, Sweden and Switzerland.

Figure 3D and Figure 4D.

*Note*: Characterized by having a farinose to minutely granular, greenish yellow thallus; long-stalked apothecia covered by a greenish-yellow pruina; a hemispherical to almost spherical capitulum with poorly developed excipulum; catenulate asci; spherical to cuboid spores 2.5–3.0 um diam. with a minutely verrucose surface (Figure 4D), 5–6 ornamentation ridges over the hemisphere. Very similar to *C. confusa* and *C. eufuracea*, for a comparison see Notes under those species

Selected specimens examined: India, Uttarkhand, 16 km NNE of Uttarkashi, between Manji and Dodi Tal, 1999, Tibell 21874 (UPS; T092 GB: PP741629); Sangam chatti, 1999, Tibell 21829 (T046, UPS, GB: PP741588, PP741615). Sweden, Jämtland. Åre par., 2.9 km WSW of Åre church, Kvarnån, 2007, Tibell 22307b (UPS, T199, GB: PP741630), 2007, Tibell 22299 (UPS; T198, GB: PP741587, PP741619). Uppland, Dannemora par., 0.5 km S of Ruddu, 2000, Tibell 22364 (UPS, epitype; GB: AF445357); Vänge par., Fiby urskog, 2020, Temu 442 (SGT 442; GB: PP741586, PP741618). Switzerland, no further locality data (GB: KX098351).

## 4. Discussion

Here we have emended Acharius’ description of *Coniocybe* to also include the occurrence of catenulate asci and a very unusual type of spore micro-ornamentation consisting of short irregularly arranged ridges. Along with molecular data, this has led to the exclusion of some species originally included in the genus and we have also been able to add two species unknown to Acharius, one of them, *C. eufuracea*, a new species.

It is interesting that Acharius’ recognition of *Coniocybe* stands up quite well to scrutinizing by genetic investigations some 200 years later, insofar that both *C. brachypoda* and ‘*C. furfuracea*’ (although this species in Acharius’ sense might just as well have been *C. eufuracea*) were included, and the features of these species very much put their mark on the generic description. This emphasized the farinose thallus, the long, thin and flexuous stalks, the small, spherical capitulum with its knob-like central part, a rather pale mazaedium, and the occurrence of a pruina. He also commented that the species occur in dark and humid sites. In Acharius’ own words (in Swedish): ‘*Ehuru vid första påseende en visss formal likhet visar sig imellan detta och nyss förut beskrifna Slägtes* (i.e., *Calicium*) *arter, så upptäckas dock snart vid en nogare uppmärksamhet den väsendtliga skillnad, som är dem imellan*…’—in short, careful attention convinced Acharius about the considerable difference between *Coniocybe* as compared to *Calicium*. Yes, we have to acknowledge Acharius’ careful attention! However, apart from *C. brachypoda* and ‘*C. furfuracea*’, Acharius also included ‘*C. stilbea*’ = *Sclerophora pallida* (Pers.) Y.J. Yao & Spooner and *Calicium aciculare* Ach. = *Chaenotheca hispidula* (Ach.) Zahlbr in *Coniocybe*. Both these latter species, although different from *Calicium* in having a pale brown mazaedium, rather poorly agree with Acharius’ description of *Coniocybe*. The inclusion of *C. gracilenta* Ach. is, in contrast, more excusable since, in its ascoma morphology, it is quite similar to that of *Coniocybe*, but DNA information is at odds with this classification and has shown that, although it belongs in *Chaenotheca* s. lat., it is outside Coniocybe (Figure 1).

## Figures and Tables

**Table 1:** Species and GenBank accession numbers of sequences used in the DNA analyses. Newly produced sequences in bold.

Species	Isolation	Country	Voucher	GB Acc. No		
				**ITS**	**LSU**	***rpb1***
*Chaenotheca* *biesboschii*	L380	Netherlands	A.v.d.Pluijm3244 (UPS)	MK514539	**PP741614**	**PP750714**
*Ch. brunneola* 1	T076	Sweden	Tibell22202 (UPS)	AF297964	**PP741600**	⸺
*Ch. brunneola* 2	T193	Estonia	TU<EST>: 76415	KX348127	**PP741602**	⸺
*Ch. chlorella*	T061	Sweden	Tibell22186	AF297965	**PP741610**	⸺
*Ch. ferruginea* 1	T099	Sweden	Tibell22276 (UPS)	MK514541	**PP741603**	⸺
*Ch. ferruginea* 2	DF82/T835	Switzerland	WSL: DF82	KX098349	**PP741604**	⸺
*Ch. gracilenta* 1	T055	Sweden	Tibell22197 (UPS)	AF410675	**PP741612**	**PP750720**
*Ch. gracilenta* 2	T135/T310	Sweden	Thor (hb. Thor)	AF410676	**PP741613**	⸺
*Ch. gracillima* 1	T037	Sweden	Tibell17052 (UPS)	AF298127	**PP741605**	**PP750721**
*Ch. gracillima* 2	T107	New Zealand	Tibell16725 (UPS)	AF408682	**PP741611**	**PP750722**
*Ch. hygrophila*	T024	Japan	Thor 15612 (UPS)	AF298129	**PP741601**	⸺
*Ch. trichialis* 2	UPSC: 2297/ T038	Sweden	Tibell16878 (UPS)	AF298139	KF157985	**PP750723**
*Ch. trichialis* 1	⸺	⸺	Prieto3028 (S)	JX000102	JX000085	JX000136
*Ch. trichialis* 3	T129	Sweden	Tibell22300 (UPS)	AF421203	**PP741606**	**PP750724**
*Ch. xyloxena* 1	T066	Sweden	Tibell22188 (UPS)	AF298140	**PP741608**	**PP750725**
*Ch. xyloxena* 3	T181/T131	Sweden	Tibell22329 (UPS	AF421212	**PP741609**	**PP750727**
*Ch. xyloxena* 2	T103	Sweden	Tibell22171 (UPS)	AF421210	**PP741607**	**PP750726**
*Coniocybe* *brachypoda* 3	T030	Sweden	Tibell17062 (UPS) /UPSC2446	AF297962	⸺	⸺
*C. brachypoda* 1	T060/ Prieto3023	Sweden	Tibell22193(UPS) /Prieto3023 (S)	AF297963	JX000086.1	JX000135
*C. brachypoda* 2	T027	New Zealand	UPSC2070; Tibell16627	**PP741625**	⸺	⸺
*C. confusa* 1	C1	Australia	Kantvilas280/19(Ho)	**PP741626**	⸺	⸺
*C. confusa* 2	C2	Australia	Kantvilas280 /19 (Ho)	**PP741627**	⸺	⸺
*C. eufuracea* 10	T036	Japan	Thor15698	AF298124	⸺	⸺
*C. eufuracea* 3	T081/T062	Sweden	Tibell22190 (UPS)	AF298125	**PP741624**	**PP750718**
*C. eufuracea* 1	⸺	⸺	Wedin6366 (UPS)	NR120128_1	JX000087	JX000137
*C. eufuracea* 8	T439	Canada	Koffman387	**PP741591**	**PP741620**	⸺
*C. eufuracea* 4	SGT422	Tanzania	Temu422	**PP741593**	**PP741622**	**PP750715**
*C. eufuracea* 2	SGT443	Sweden	Temu443	**PP741592**	**PP741623**	**PP750717**
*C. eufuracea* 5	T901	India	Tibell25106	**PP741594**	**PP741621**	**PP750719**
*C. eufuracea* 9	T355	India	Tibell23224	**PP741628**	⸺	⸺
*C. eufuracea* 6	SGT426	Tanzania	Temu426	**PP741589**	**PP741616**	⸺
*C. eufuracea* 7	SGT431	Tanzania	Temu431	**PP741590**	**PP741617**	**PP750716**
*C. furfuracea* 1	SGT442	Sweden	Temu442	**PP741586**	**PP741618**	⸺
*C. furfuracea* 2	T046	Sweden	Tibell21829	**PP741588**	**PP741615**	⸺
*C. furfuracea* 3	T198	Sweden	Tibell22299	**PP741587**	**PP741619**	⸺
*C. furfuracea* 4	T155	Sweden	Tibell22364 (UPS)	AF445357	⸺	⸺
*C. furfuracea* 5	T092	India	Tibell21874	**PP741629**	⸺	⸺
*C. furfuracea* 6	WSL:DF252	Switzerland	WSL:DF252	KX098351_1	⸺	⸺
*C. furfuracea* 7	T199	Sweden	Tibell22307b	**PP741630**	⸺	⸺
*Sclerophora coniophaea*	⸺	⸺	Wedin6367 (UPS)	⸺	JX000094	JX000145
*S. farinacea*	⸺	Estonia	Wedin6414 (UPS)	JX000113	JX000095	JX000144

A “⸺” sign indicates missing data.

## Data Availability

All new sequence data in the manuscript marked with GenBank accession numbers in bold are available in NCBI.

## Supplementary Materials

The following supporting information can be downloaded at: https://www.mdpi.com/article/10.3390/jof10050363/s1, Figure S1: ITS phylogeny; Figure S2: LSU phylogeny; Figure S3: RPB1 phylogeny.
