# Supplementary figures and images for: Coniocybe Ach. Revisited
Source: J Fungi (Basel). 2024 May 20;10(5):363. doi: 10.3390/jof10050363 (PMC11122650; doi:10.3390/jof10050363)

Figure S1: ITS phylogeny

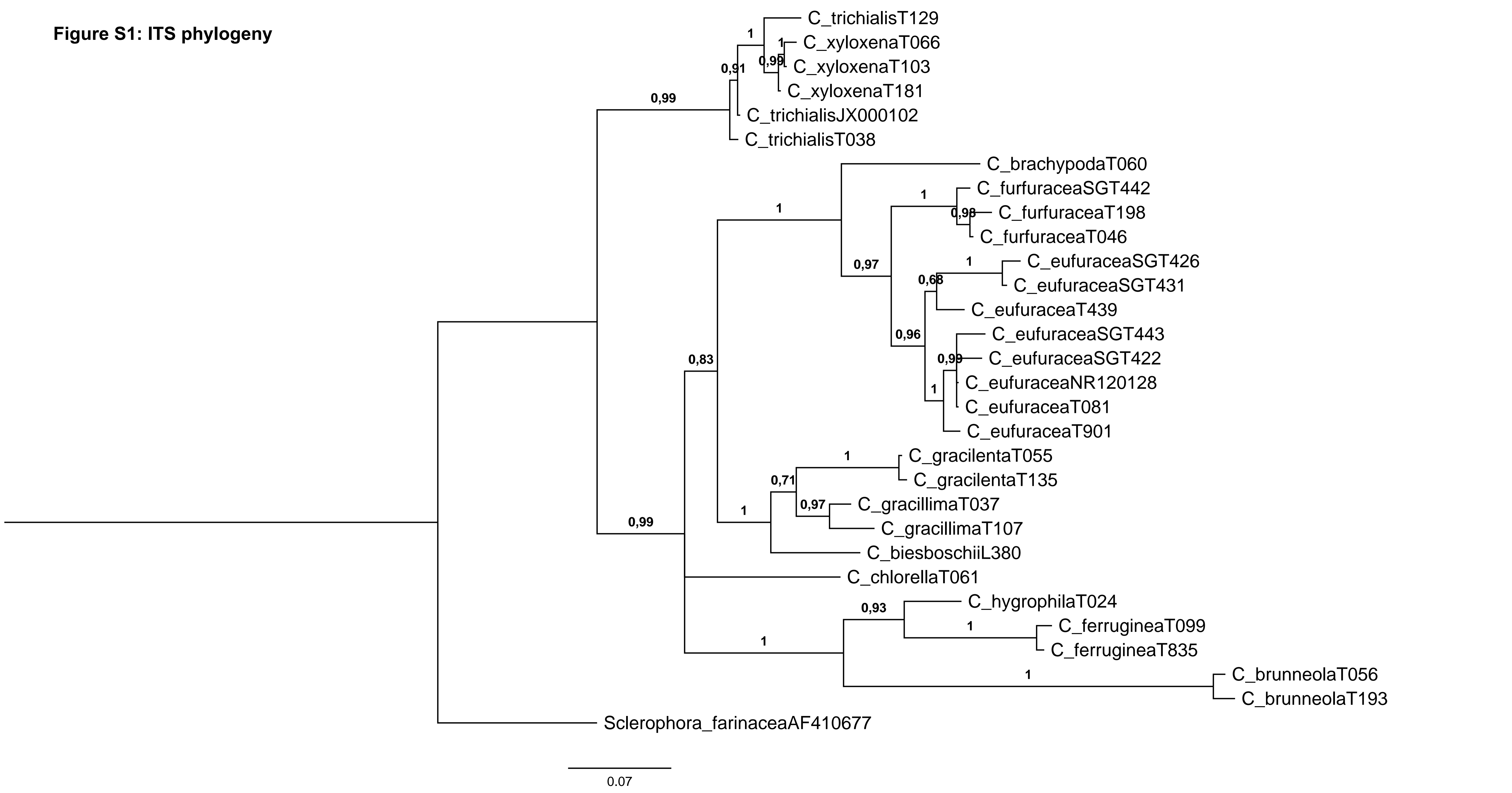

Figure S2: LSU phylogeny

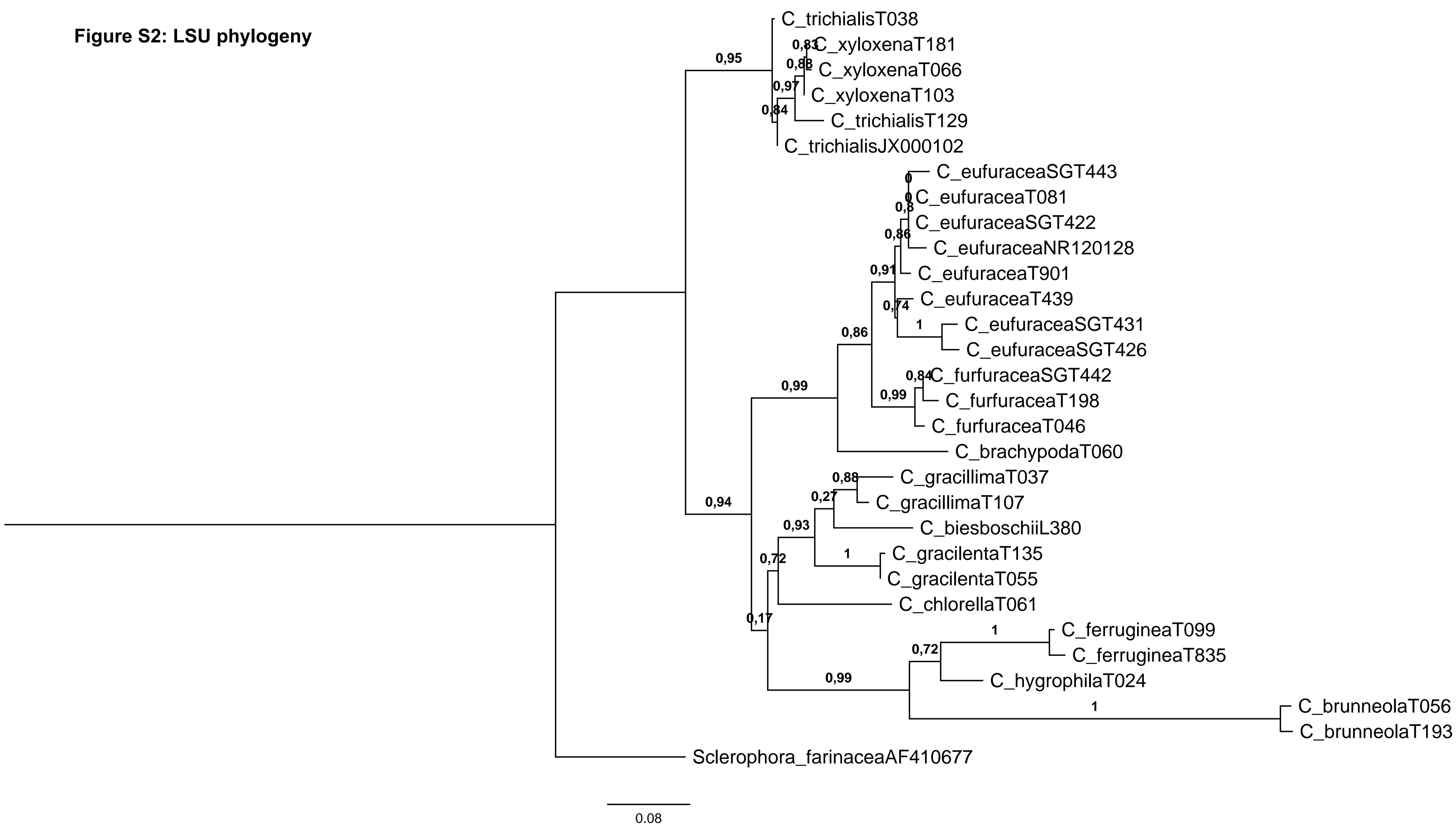

Figure S3: RPB1 phylogeny

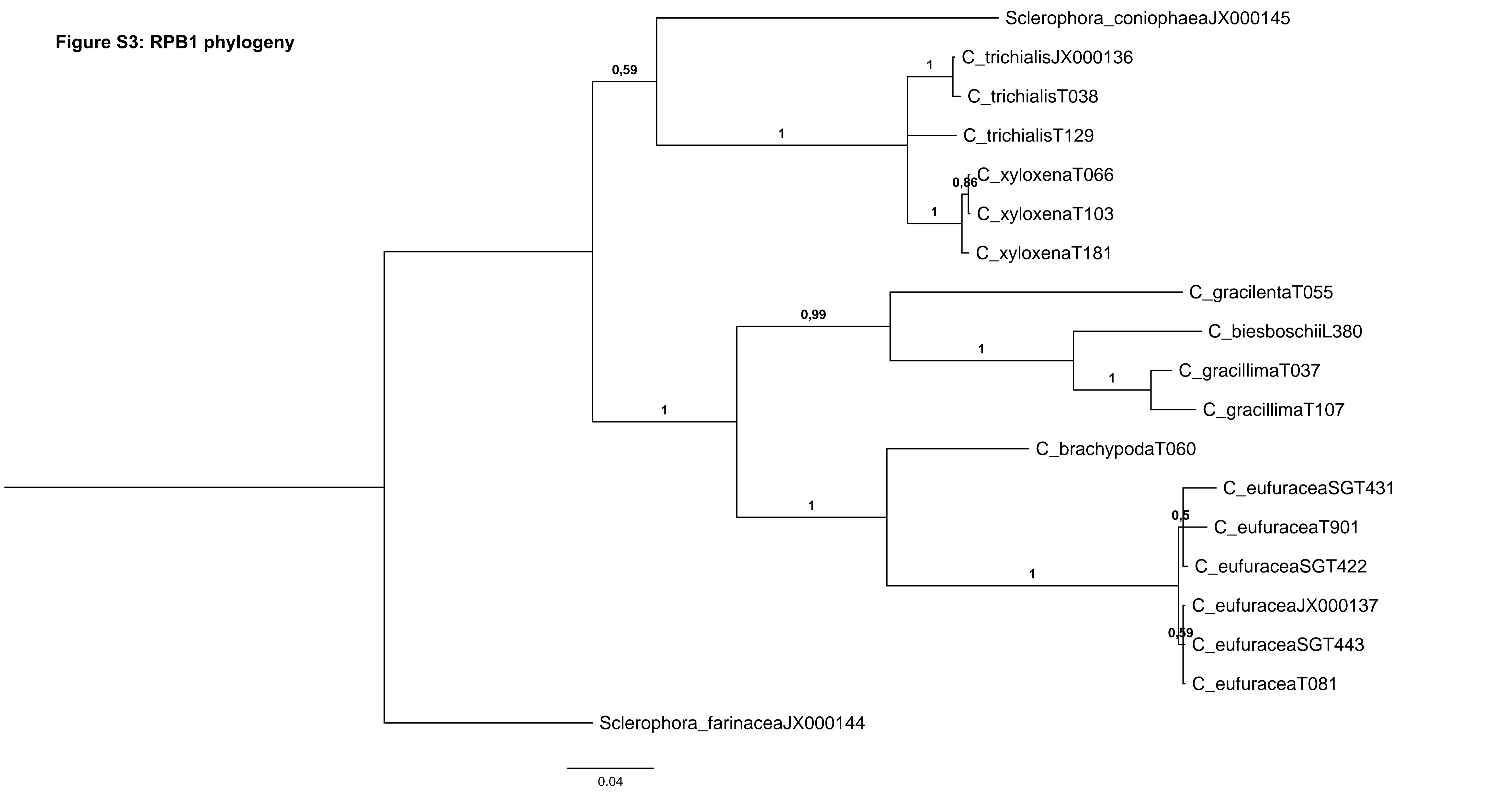

Supplement: Supplementary file 1 [file jof-10-00363-s001.zip › jof-2957781-supplementary.pdf]
